# Southern Dental Association

**Published:** 1885-06

**Authors:** 


### Southern Dental Association.

The annual meeting of the Southern Dental Association was held in New Orleans, March 31st and the three succeeding days. The meeting was the best ever held by this body; there was a larger number in attendance and more work was done than ever before at one meeting. This number of the REGISTER contains a full and complete report of the proceedings, except that of the papers read, a careful synopsis is given instead of the full paper. This report excludes almost everything else from this number, but the interested reader will much prefer this rather than to have it in fragments running through three or four months. The report will well repay a careful perusal, as it contains the views of some of the best men in the profession on some important subjects.

